# Link of TMPRSS2 expression with tumor immunogenicity and response to immune checkpoint inhibitors in cancers

**DOI:** 10.1186/s12967-025-06177-z

**Published:** 2025-03-07

**Authors:** Karthikeyan Subbarayan, Helena Bieber, Chiara Massa, Felipe Adonis Escalona Rodríguez, SM Al Amin Hossain, Lisa Neuder, Wafa Wahbi, Tuula Salo, Sandy Tretbar, Ahmed Al-Samadi, Barbara Seliger

**Affiliations:** 1 Medical Faculty, Martin Luther University Halle-Wittenberg, Halle (Saale), Germany; 2 Institute of Translational Immunology, Faculty of Health Sciences, Brandenburg Medical School “Theodor Fontane”, Brandenburg an der Havel, Germany; 3 Center for Protein Studies, Faculty of Biology, University of Havana (UH), Havana, Cuba; 4 NanoCancer, Molecular Immunology Center (CIM), Havana, Cuba; 5 Department of Oral and Maxillofacial Diseases, Clinicum, University of Helsinki, Helsinki, Finland; 6 Cancer and Translational Medicine Research Unit, University of Oulu, Oulu, 90014 Finland; 7 Fraunhofer Institute for Cell Therapy and Immunology, Leipzig, Germany; 8 Institute of Dentistry, School of Medicine, Faculty of Health Sciences, University of Eastern Finland, Kuopio, Finland

**Keywords:** SARS-CoV-2, TMPRSS2, Immune escape, Immune response, Tumors

## Abstract

**Background:**

SARS-CoV-2 and other viruses rely on the protease function of the TMPRSS2 protein to invade host cells. Despite cancer patients often experience poorer outcomes following SARS-CoV-2 infection, the role of TMPRSS2 in different cancer types has not yet been analyzed in detail. Therefore, the aim of the study was to determine the expression, function and clinical relevance of TMPRSS2 in tumors.

**Methods:**

Publicly accessible RNA sequencing data from tumors, adjacent tissues and whole blood samples of COVID-19 patients as well as data from human tumor epithelial and endothelial cells infected with SARS-CoV-2 were analyzed for TMPRSS2 expression and correlated to the expression of immune-relevant genes and clinical parameters. In vitro models of cells transfected with TMPRSS2 (TMPRSS2^high^), siTMPRSS2 or mock controls (TMPRSS2^low^ cells) were analyzed by qPCR, flow cytometry, ELISA and Western blot for the expression of immune response-relevant molecules. Co-cultures of TMPRSS2 model systems with blood peripheral mononuclear cells were employed to evaluate immune cell migration, cytotoxicity and cytokine release.

**Results:**

Higher expression levels of TMPRSS2 were found in blood from patients infected with SARS-CoV-2, while TMPRSS2 expression levels significantly varied between the tumor types analyzed. TMPRSS2^high^ tumor cells exhibit increased activity of the interferon (IFN) signal pathway accompanied by an increased expression of class I human leukocyte antigens (HLA-I) and programmed cell death ligand 1 (PD-L1) elevated interleukin 6 (IL-6) secretion and reduced NK cell-mediated cytotoxicity compared to TMPRSS2^low^ mock controls. Treatment with a Janus kinase (JAK) 2 inhibitor or TMPRSS2-specific siRNA decreased TMPRSS2 expression. Co-cultures of the in vitro TMPRSS2 models with peripheral blood mononuclear cells in the presence of the immune checkpoint inhibitor nivolumab resulted in a significantly increased migration and infiltration of immune cells towards TMPRSS2^high^ cells and a reduced release of the innate immunity-related cytokines CCL2 and CCL3.

**Conclusions:**

This study provides novel insights into the role of TMPRSS2 in various tumor systems and the impact of SARS-CoV-2 infection on the host immunogenicity via the activation of immune-relevant pathways. These findings were linked to the efficacy of immune checkpoint inhibitor therapy, offering a potential alternative strategy to mitigate the severity of COVID-19.

**Supplementary Information:**

The online version contains supplementary material available at 10.1186/s12967-025-06177-z.

## Background

Coronavirus disease 2019 (COVID-19) has rapidly emerged as a global health pandemic mediated by infection with the severe acute respiratory syndrome coronavirus 2 (SARS-CoV-2). The virus utilizes the angiotensin-converting enzyme 2 (ACE2) receptor for its cellular entry and the transmembrane protease serine 2 (TMPRSS2) for the priming of the spike (S) protein [1]. Since some coronaviruses, such as SARS-CoV-1, MERS-CoV, and SARS-CoV-2, are activated by TMPRSS2 [2], there is an urgent need for the development of broad anti-viral strategies targeting multiple coronaviruses by focusing on common elements, such as TMPRSS2 and ACE2. In this context, TMPRSS2 inhibitors have been shown to block viral infections [3] suggesting their use as a potential therapeutic approach [4, 5].

Moreover, there exists growing evidence that tumor patients have an increased risk of COVID-19 infection and are more susceptible to severe illness with a worse outcome [6–12]. So far, there exists only limited information about the link between the expression of immune-relevant molecules in cancer patients, susceptibility to SARS-CoV-2 infection and the severity of the disease [13]. For example, an upregulation of the programmed cell death ligand (PD-L)1 expression during the cytokine storm of SARS-CoV-2-infection has been detected [14], but also infections with other viruses have been shown to upregulate PD-L1 expression, leading to immune escape [14, 15]. Consequently, PD-L1/PD-1 immune checkpoint inhibitor (ICPi) therapy might represent a potential treatment option to enable T cells to combat cancer cells [16], while controlling SARS-CoV-2 infection.

Under physiological conditions, TMPRSS2 is expressed in various tissues, including pancreas, prostate, stomach, kidney, small intestine as well as the respiratory tract, which is a primary site of SARS-CoV-2 infection [17]. The TMPRSS2 expression in pan-tissues is associated with pathways involved in immune metabolism, cell growth as well as stromal and cancer signatures [18]. During pathophysiological conditions, the TMPRSS2 expression levels and their prognostic value highly varied across different cancer types [17]. For example, TMPRSS2 levels are elevated in prostate cancer [19], but decreased in colon cancer when compared to corresponding normal tissues [17]. Furthermore, the expression of TMPRSS2 in lung adenocarcinoma is linked to a positive prognosis, while it is associated with a negative prognosis in breast cancer [20].

Cancer patients may have a higher mortality rate due to a COVID-19-mediated hyperinflammatory state, which is accompanied by severe disease manifestations [8, 14]. In severe cases, virus spread is linked to excessive IL-6 production associated with severe inflammation and tissue damage, while this response is controlled in mild cases [14, 21]. In contrast, IL-2 has the potential to improve the immune response to COVID-19 but is also a key factor in disease severity due to its involvement in cytokine release and immune regulation [22].

The JAK-STAT pathway is a fundamental signaling mechanism involved in anti-viral and anti-tumoral immune responses [23], but also involved in the hyperinflammatory response occuring in severe COVID-19 cases [24]. However, the impact of SARS-CoV-2-associated molecules, including TMPRSS2, on the JAK-STAT signal transduction has not yet been determined. Therefore, an increased understanding of the functions and interactions of TMPRSS2 with the JAK-STAT pathway and their regulated molecules, such as class I human leukocyte antigens (HLA-I) and PD-L1, is urgently needed and might provide insights into their potential as therapeutic targets thereby enhancing the patients’ outcomes by improving the management of COVID-19.

Consequently, this study aims to elucidate the expression patterns and clinical significance of TMPRSS2 across different cancer types by analyzing publicly available RNA sequencing data, to assess in vivo immunological pathways associated with varying TMPRSS2 expression levels and to explore how their dynamics might affect the outcome of SARS-CoV-2-infected cancer patients. These results will provide insights into potential therapeutic approaches, including immune checkpoint inhibitors (ICPi), to improve the management of cancer patients during emerging and re-emerging coronavirus outbreaks.

## Methods

### Cell culture and transfections

The human tumor epithelial cell lines MCF-7 (breast cancer (BC)), MDA-MB-468 (BC) and the endothelial cell line EA.Hy926 were obtained from the American Type Culture Collection (ATCC, Manassas, USA). All cell lines were grown in RPMI1640 medium with the exception of EA.Hy926, which was cultured in DMEM/F12. The culture mediums were supplemented with 2 mM L-glutamine, 10% fetal calf serum (FCS, PAN-Biotech, Aidenbach, Germany) and appropriate antibiotics.

Cell lines were transfected with the TMPRSS2 expression vector, designated as TMPRSS2^high^ (Addgene, Watertown, USA), using the Effectene Transfection Reagent (Qiagen, Hilden, Germany) according to the manufacturer’s instructions. A control was established using a mock vector, referred to as TMPRSS2^low/mock^.

Lipid nanoparticles (LNPs) were formulated by mixing lipid stock solutions in ethanol, composed of D-Lin-MC3-DMA, DPPC, Cholesterol, and PEG-DMG in a molar ratio of 50:10:35:5. These were combined with either a siTMPRSS2 or negative control (Thermo Fisher, Waltham, USA), utilizing an amine-to-phosphate (N/P) ratio of 3.1, which corresponds to 0.054 µg of nucleic acid per µmol of total lipid. Subsequently, the LNP complex was used to transfect the TMPRSS2^high^ MDA-MB-468 cells, resulting in the generation of TMPRSS2-silenced cells.

### RNA extraction and real-time quantitative RT-PCR

Cellular RNA was extracted using the NucleoSpin RNA II kit (Macherey-Nagel, Düren, Germany). 2 µg RNA/ sample was converted into cDNA using the Revert H Minus First Strand cDNA synthesis kit (Fermentas, Thermo Fisher) and oligo(dT)18 primer following the manufacturer’s instructions. Utilizing a standard laboratory protocol, qRT-PCR was conducted on a Bio-Rad CFX96 system (Bio-Rad, Hercules, USA) with the platinum SYBRGreen qPCR Supermix UDG (Thermo Fisher) as described [25] using primers directed against HLA-I, APM components, IFN signaling molecules, PD-L1 and TMPRSS2-regulated genes (Supplementary Table 1). Data were evaluated using the comparative quantification mode of the CFX Maestro Software 2.3 (Bio-Rad) and presented as a mean of a minimum of three independent experiments.

### Western blot analysis

For Western blot analysis, 30 µg protein/ sample was separated by SDS-PAGE and then transferred using the iBlot™ 2 Dry Blotting System (Invitrogen, Thermo Fisher) prior to staining with antibodies (Abs) specific to TMPRSS2, IFI27 and STAT1 (Cell Signaling Technology, Danvers, USA). To ensure equal protein loading, the blot was stained with a monoclonal antibody (mAb) against ACTB (Cell Signaling Technology, Danvers, USA). A horse-reddish peroxidase (HRP) conjugated secondary Ab was applied before visualizing the proteins using an ECL-based system for chemiluminescence.

### Flow cytometry

Flow cytometry was conducted as recently described [26]. The antibodies employed for flow cytometry were HLA-I and PD-L1 mAbs (Invitrogen, Waltham, USA). Briefly, tumor cells were labeled with the appropriate amounts of antibodies at 4 °C in darkness for 30 min. HLA-I and PD-L1 expression was assessed on a NAVIOS flow cytometer (Beckman Coulter, Brea, USA) and data was analyzed using the Kaluza Software. The results were presented as mean fluorescence intensity (MFI) from at least three independent experiments.

### Quantification of IL-6 by ELISA

To quantify IL-6 in TMPRSS2^low^ and TMPRSS2^high^ EA.Hy926 cells, the supernatants were collected, and the amount of IL-6 produced by the endothelial cells was calculated using an ELISA (LEGENDplex™ Macrophage/Microglia Panel, BioLegend) following the manufacturer’s instructions. Data acquisition was conducted using the BD Symphony A3, and analysis was performed with the LEGENDplex™ Data Analysis Software Suite.

### mRNA sequencing and data analyses

RNA sequencing was performed on the Illumina Noveseq 6000 platform by Novogene (Cambridge, United Kingdom). The reference genome and gene model annotation files were obtained from the genome website (NCBI/UCSC/Ensembl). HTSeq v0.6.1 was used to quantify gene expression levels, and the FPKM of each gene was calculated based on the gene’s length and the mapped read counts [27]. The initial analysis of differential gene expression (DGE) between TMPRSS2^low^ and TMPRSS2^high^ MCF7 and EA.Hy926 cells using the DESeq2 R package (2_1.6.3) was conducted by Novogene (Munich, Germany). The p-values were adjusted using Benjamini and Hochberg’s approach to control the False Discovery Rate (FDR). Genes with an adjusted p-value (Padj) < 0.05 in DESeq2 data were identified as differentially expressed. The differentially expressed genes (DEGs) were subjected to Gene Ontology (GO) and Kyoto Encyclopedia of Genes and Genomes (KEGG) enrichment analyses using the clusterProfiler (v4.0.5) R package. KEGG terms with corrected Padj value < 0.05 were considered significantly enriched.

### Microfluidic chip assay

TMPRSS2^high^ and TMPRSS2^low^ MCF-7 cells were labeled with CellTrace™ Far Red (Invitrogen, Thermo Fisher) according to the manufacturer’s instructions for microfluidic chip tests and then suspended in RPMI1640-based myogel/fibrin gel derived from human tumors containing 2.4 mg/ml myogel, 0.5 mg/ml fibrinogen (Merck, Darmstadt, Germany), 33.3 µg/ml aprotinin (Sigma-Aldrich) and 0.3 U/ml thrombin (Sigma-Aldrich) as well as 5 µM of IncuCyte caspase-3/7 green (Sartorius, Göttingen, Germany) to identify apoptotic cells. The TMPRSS2^high^ and TMPRSS2^low^ MCF-7 cells were then divided into a control group without drug and a group treated with 0.5 µM nivolumab (Opvido^®^, Selleckchem, Houston, Texas, USA). Subsequently, 2 µL of cell suspension (i.e., 500 cells) were loaded into separate small “cancer cell channels” of the microfluidic chip as previously described [28].

Peripheral blood mononuclear cells (PBMNCs) purified by gradient density centrifugation from the buffy coats of healthy donors provided by the Finnish Red Cross were stained with CellTrace™ Violet (Invitrogen) following the manufacturer’s instructions. Cell viability and number were assessed using trypan blue staining with CellCountess (Invitrogen). After staining, the cells were suspended in cell culture media supplemented with 10 ng/ml of recombinant human IL-2 (BioLegend, San Diego, California, USA) and 5 µM caspase-3/7 green (Sartorius). The PBMNCs were then divided into a control group without drug and a group treated with 0.5 µM nivolumab. Subsequently, 1 × 10^5^ viable PBMNCs in in 100 µL was added to the larger ‘PBMNC channels’ of the chip as previously described [28]. In controls without PBMNCs, 100 µL of cell culture media containing 5 µM caspase-3/7 green was injected.

Following the injections, the chips were placed in a cell culture incubator and incubated for 72 h with daily imaging conducted using a Nikon Ti-E with Alveole Primo microscope (Nikon, Tokyo, Japan) connected to a Hamamatsu Orca Flash 4.0 LT B&W camera (Hamamatsu Photonics, Hamamatsu, Japan). The conditioned medium was then collected from the chips and stored at -80 °C until further use.

### Cytokine profiling

The Abcam FirePlex Service (Boston, USA) was used to perform cytokine profiling on the conditioned media from the microfluidic chips with a FirePlex^®^-96 Key Cytokines (Human) Immunoassay Panel (Abcam, Cambridge, UK). This panel can detect 17 cytokines, including the granulocyte-macrophage colony-stimulating factor (CSF2, GM-CSF), IL-1B, IL-2, IL-4, IL-5, IL-6, IL-9, IL-10, IL-12 A, IL-13 and IL-17 A, CXCL8, IFNγ, monocyte chemoattractant protein-1 (MCP-1, CCL2), macrophage inflammatory protein 1 alpha (MIP1-α, CCL3), macrophage inflammatory protein 1 beta (MIP1-β, CCL4) and tumor necrosis factor (TNF)-α. Each sample was analyzed in duplicate.

### NK cell activity

Human PBMNCs were activated for 18 h using 1 ng/ml of IL-12, 5 ng/ml of IL-15 (Immunotools, Friesoythe, Germany) and 50 ng/ml of IL-18 (Biovision, Milpitas, CA, USA) in X–vivo15 medium (Lonza). Subsequently, the cells were co-cultured with TMPRSS2^high^ and TMPRSS2^low^ target cells at a ratio of 1:1 followed by degranulation assay. Briefly, an anti-CD107a antibody was added for 1 h to the co-culture before the cells were stained with monoclonal antibodies (mAbs) against CD3, CD16 and CD56 (BioLegend) after 4 h to identify NK cells and assess overall NK cell activity.

### Multi-dataset analysis of transcriptomic and genomic data

Transcriptomic data from 24 healthy controls and 62 COVID-19 patients [29, 30] were examined using COVID19db (ID: COVID000010). Previously published characteristics of the patients, such as age, gender and blood parameters, were referenced [29]. Data from Calu3 cells, a lung adenocarcinoma cell line infected with SARS-CoV-2, was obtained from GEO: GSE147507. Metadata from a cohort of SARS-CoV-2 and other respiratory viruses, including human parainfluenza virus 3 (HPIV3), respiratory syncytial virus (RSV), mutant influenza A virus (IAVdNS) infected cells as well as COVID-19 positive lung biopsies (GEO accession: GSE147507) were analyzed using ImmGen of Immunological Genome Project [31–33]. Single-cell RNA-seq data of peripheral blood from patients with severe COVID-19 were obtained from ImmGen [34]. Additional datasets were obtained from the Gene Expression Omnibus (GEO), which included the following: an organoid model infected with the SARS-CoV-2 Alpha variant (B.1.1.7) (GEO: GSE178333), samples from patients experiencing long COVID-19-a condition marked by persistent symptoms following SARS-CoV-2 infection (GEO: GSE224615; [35]) and samples from C57BL/6J wild-type mice infected with SARS-CoV-2 (GEO: GSE253635; [36]). The expression of the TMPRSS2 transcript across different tumor types and their corresponding normal tissues was analyzed using The Cancer Genome Atlas (TCGA) datasets through UALCAN [37, 38]. Metadata from a cohort of BC (*n* = 782) and lung cancer (*n* = 91) cases obtained from TCGA (portal: https://portal.gdc.cancer.gov) [39] and GEO dataset (GSE18842) were analyzed using the R2: Genomics analysis and visualization platform (http://r2.amc.nl). The gene expression patterns of TMPRSS2, HLA class I APM, IFN pathway components and PD-L1 were extracted from the datasets.

### Statistical analysis

The graphical presentations, Student’s t-test and one-way ANOVA were conducted using Microsoft Excel-Office 365, BioRender and R (RStudio 3.0). Analysis of cytokine release was performed using the FirePlex™ Analysis Workbench software (https://www.abcam.com/kits/fireplex-analysis-workbench-software). A p-value < 0.05 was considered statistically significant (*, *p* < 0.05; **, *p* < 0.01; ***, *p* < 0.001).

## Results

### High levels of TMPRSS2 expression following SARS-CoV-2 infection

**Fig. 1:**
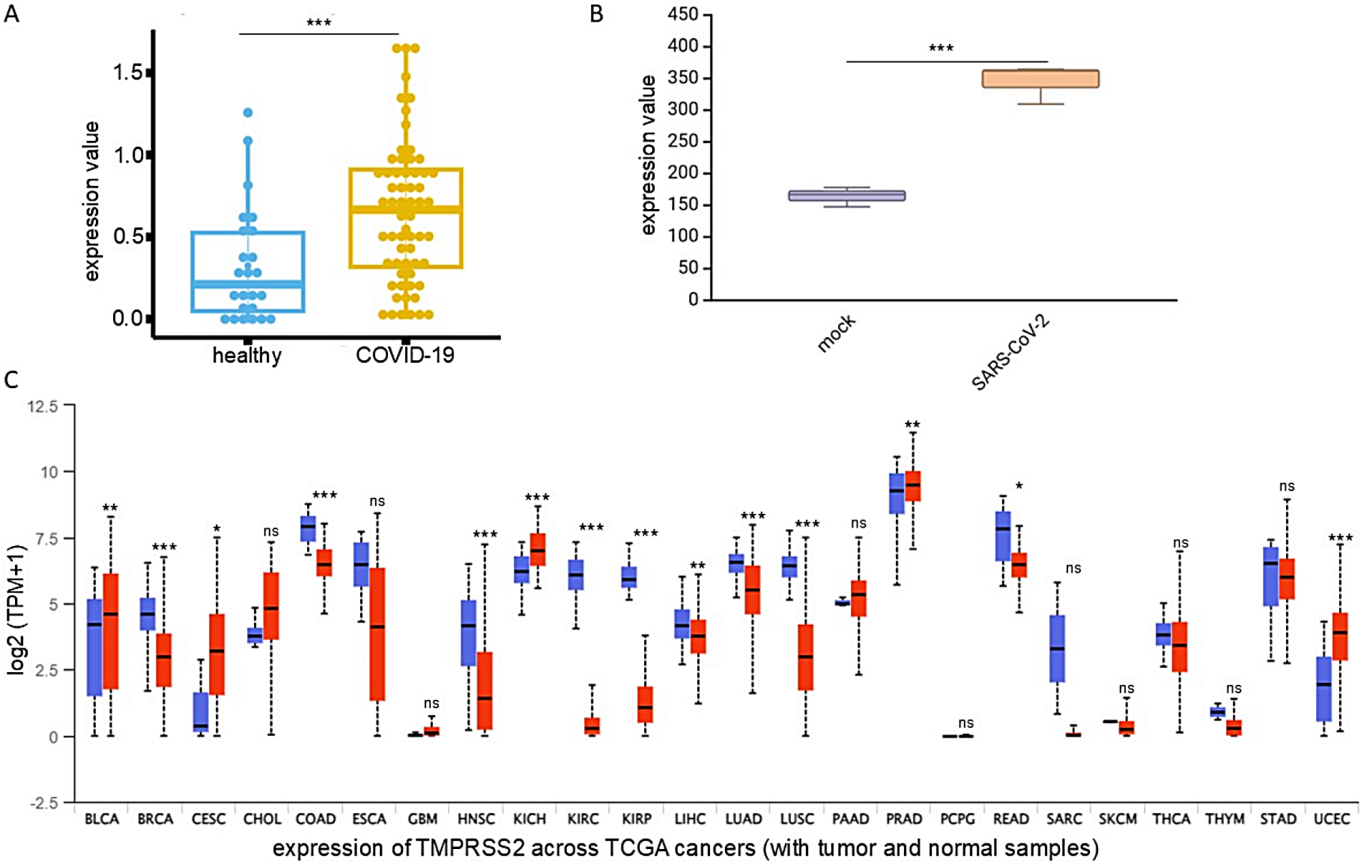
Increased levels of TMPRSS2 expression in blood samples from COVID-19 patients and in cell lines infected with SARS-CoV-2 compared to uninfected controls. **A**: TMPRSS2 expression levels were analyzed in blood samples from 24 healthy individuals and 62 patients with COVID-19. These data were obtained from whole blood transcriptomics analyses (COVID19db). **B**: TMPRSS2 expression in Calu3 lung carcinoma epithelial cells was evaluated after infection with SARS-CoV-2 (GEO: GSE147507). **C**: The TMPRSS2 transcriptomic expression across various tumor types (red bars) and their adjacent normal tissues (blue bars) was examined using TCGA datasets. The results are displayed in a bar chart. t.test, ns: *p* > 0.05; *: *p* ≤ 0.05; **: *p* ≤ 0.01; ***: *p* ≤ 0.001

RNAseq data from PBMNCs collected from 24 healthy individuals and 62 COVID-19 patients (COVID19db ID: COVID000010) revealed increased levels of TMPRSS2 mRNA in PBMNCs after SARS-CoV-2 infection (Fig. 1A). This increase was comparable to the TMPRSS2 mRNA levels observed in SARS-CoV-2-infected Calu3 lung carcinoma cells (Fig. 1B). Analyses of TCGA datasets showed a heterogeneous expression of TMPRSS2 across different cancer types. When compared to corresponding normal tissue, higher mRNA expression levels of TMPRSS2 were found in BLCA, CESE, KICH, PRAD and UCEC, lower in BRCA, COAD, HNSC, KIRC, KIRP, LIHC, LUAD, LUSC and READ whereas TMPRSS2 expression was unchanged in CHOL, ESCA, GBM, PAAD, PCPG, SARC, SKCM, THCA, THYM and STAD (Fig. 1C).

### Influence of TMPRSS2 overexpression on transcriptomic profiles

**Fig. 2:**
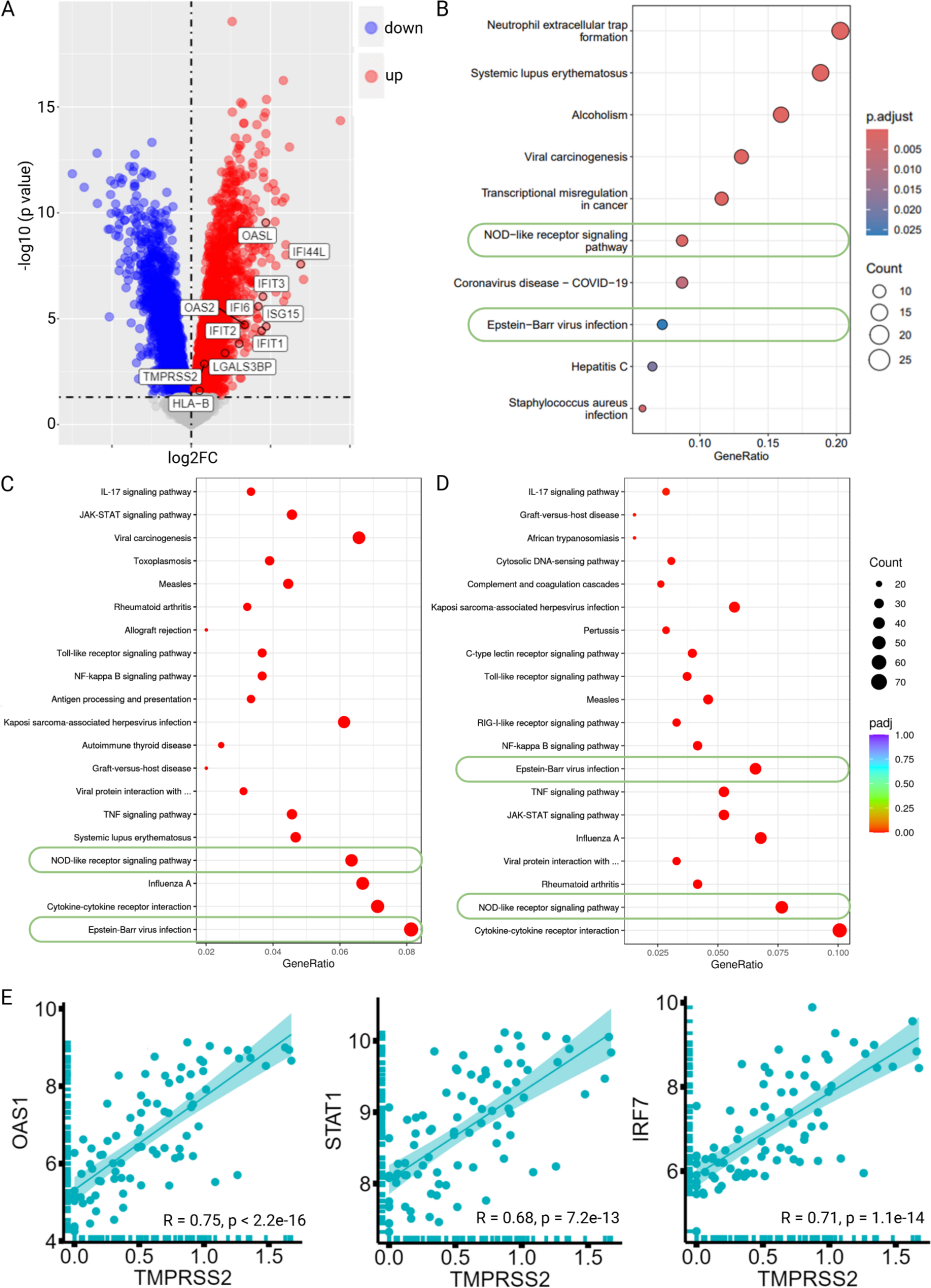
An analysis of transcriptional profiles and KEGG pathway enrichments in COVID-19 vs. healthy PBMNCs along with TMPRSS2^high^ versus TMPRSS2^low^ MCF-7 and EA.Hy926 cells were conducted using RNA sequencing. **A**: A Volcano plot depicts the differentially expressed genes (DEGs) between COVID-19 and healthy blood samples (COVID19db). Genes that are significantly downregulated are indicated in dark blue, while significantly upregulated genes are shown in red and non-significantly regulated genes are represented in grey. **B**: The top 10 enriched KEGG pathways from the upregulated genes between COVID-19 and healthy blood samples are presented. **C**, **D**: The top 20 enriched KEGG terms derived from the upregulated genes of TMPRSS2^high^ versus TMPRSS2^low^ MCF-7 (**C**) and EA.Hy926 (**D**) cells are displayed. The enriched GO terms found in both TMPRSS2^high^ and COVID-19 are highlighted with green circles. **E**: Correlation plots illustrate the commonly upregulated genes in the two most enriched KEGG pathways concerning TMPRSS2 expression from blood samples of COVID-19 patients (COVID19db). The gene expressions of OAS1, STAT1 and IRF7 are exemplified here

To determine the effect of TMPRSS2 on the gene expression pattern, MCF-7 and EA.Hy926 were transfected with a TMPRSS2 expression vector. Global transcriptomic profiles obtained after RNA sequencing of TMPRSS2^high^ vs. TMPRSS2^low^ MCF-7 cells and EA.Hy926 demonstrated 4452 and 1949 DEGs in MCF-7 and EA.Hy926 cells, respectively. Among these DEGs, 2061 genes were upregulated and 2391 genes were downregulated in TMPRSS2^high^ MCF-7 (Supplementary Fig. 1A), while 1108 genes were upregulated and 841 genes were downregulated in TMPRSS2^high^ EA.Hy926 (Supplementary Fig. 1B). Analyses of the top 30 upregulated and downregulated genes revealed 11 commonly upregulated genes (IFI6, TMPRSS2, IFIT1, IFIT2, IFIT3, OAS2, OASL, HLA-B, ISG15, LGALS3BP and IFI44L) and only one commonly downregulated gene (PRR11) in the two cell lines. The commonly upregulated genes of the in vitro models were also upregulated in COVID-19 vs. healthy PBMNCs, as highlighted in the Volcano plot (Fig. 2A). The KEGG terms retrieved from COVID19db (Fig. 2B) and the DEGs in TMPRSS2^high^ MCF-7 (Fig. 2C) and EA.Hy926 (Fig. 2D) showed that Epstein-Barr virus infection and the NOD-like receptor signaling pathway were similarly enriched across all three settings: TMPRSS2^high^ MCF-7, TMPRSS2^high^ EA.Hy926, and COVID-19 PBMNCs. In addition, within these KEGG terms, there were 12 commonly upregulated genes, namely OAS2, OAS1, OAS3, STAT1, TNFAIP3, IRF7, STAT2, NFKBIA, IFNB1, MYD88, IRF9 and IL-6. Correlation plots between TMPRSS2 and commonly upregulated genes were representively shown for OAS1, STAT1 and IRF7 from COVID-19 vs. healthy PBMNCs (Fig. 2E). In contrast, no common KEGG terms were found in downregulated DEGs between TMPRSS2 transfectants of MCF-7 and EA.Hy926 and PBMNCs of COVID-19 patients (Supplementary Fig. 2). To expand the analysis, the upregulated genes identified in our cell culture system (MCF-7 and EA.Hy926 cells) were further investigated in an organoid model infected with the SARS-CoV-2 alpha variant (B.1.1.7) (GEO: GSE178333) and in patients with long COVID-19, a condition characterized by post-acute sequelae following SARS-CoV-2 infection (GEO: GSE224615; [35]). As shown in Supplementary Table 2, genes associated with the IFN pathway, such as IFI27, IFIT1, IFIT2, OASL and OAS3, showed significantly higher expression levels, in particular individuals with long COVID-19 when compared to non-long COVID-19 and the organoid systems infected with the alpha variant.

### Impact of TMPRSS2 on HLA-I expression, IFN signaling and NK cell recognition

**Fig. 3:**
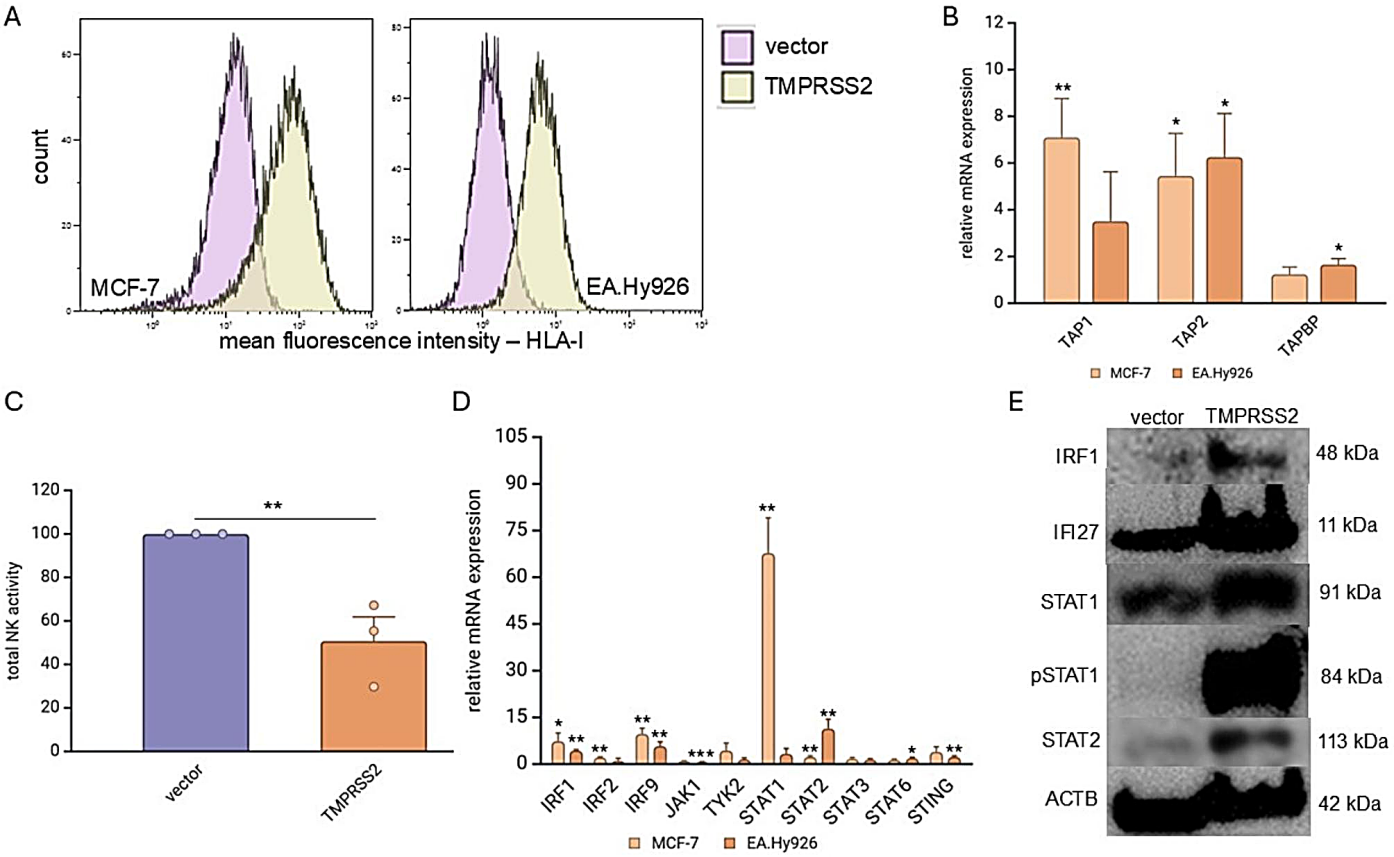
Effects of TMPRSS2 overexpression on HLA class I expression, the antigen presentation machinery (APM) and interferon (IFN) signaling in TMPRSS2-transfected cells and their influence on natural killer (NK) cell function. **A**: Flow cytometry was used to assess HLA-I surface expression in TMPRSS2 transfectants and mock control MCF-7 and EA.Hy926 cells as described in the Methods. The results are displayed as a histograms with the mean fluorescence intensity (MFI) of HLA-ABC (*n* = 3). **B**: The expression of major HLA-I APM components in TMPRSS2 transfectants was evaluated using qPCR as described in the Methods. **C**: The cytotoxic activity of NK cells from three different donors against TMPRSS2^low^ and TMPRSS2^high^ MCF-7 cells was evaluated using a CD107a degranulation assay. Data are shown as mean ± SE of CD107a degranulation upon normalization to the mock-transfected cells. **D**: TMPRSS2 transfectants were examined by qPCR for the expression of components of the IFN as well as JAK-STAT signaling pathways. The results are shown as an x-fold induction in the expression of these signaling components in TMPRSS2 transfectants relative to mock controls. **E**: Representative Western blot analyses of TMPRSS2^high^ and mock-transfected using antibodies directed against IRF1, IRF27, STAT1, pSTAT1 and STAT2. Staining with an anti-ACTB antibody served as a loading control

Since the RNAseq data revealed an increased expression of IFN signaling genes in TMPRSS2^high^ vs. TMPRSS2^low^ cells, the impact of TMPRSS2 on the expression of HLA-I surface antigens was determined in MCF-7 and EA.Hy926 cells by flow cytometry. As shown in Fig. 3A, overexpression of TMPRSS2 resulted in an upregulation of HLA-I surface antigens, which could be correlated to enhanced mRNA expression of major components of the antigen processing machinery (APM) such as the transporter associated with antigen processing (TAP)1, TAP2 and TAPBP were upregulated by TMPRSS2 overexpression (Fig. 3B). As expected, the TMPRSS2-mediated HLA-I upregulation had also an impact on NK cell responses. Using a CD107 degranulation assay, a reduced NK cell recognition of TMPRSS2^high^ compared to TMPRSS2^low^ MCF-7 cells was identified (Fig. 3C).

Based on the KEGG pathway enrichment analysis, different JAK-STAT signaling molecules, as well as IFN pathway genes, were also investigated in TMPRSS2^high^ vs. TMPRSS2^low^ tumor cells. A significant increase in the mRNA expression of IRF1, IRF9, STAT1, STAT2 and STING in TMPRSS2^high^ transfectants compared to the TMPRSS2^low^ mock controls was found (Fig. 3D). The TMPRSS2-mediated altered mRNA levels were accompanied by increased protein levels as representatively shown for IRF1, IFI27, STAT1, pSTAT1 and STAT2 in TMPRSS2^high^ vs. TMPRSS2^low^ cells (Fig. 3E).

The link between TMPRSS2 expression and immune response-relevant profiles was further validated by in silico analyses of cancer genome databases with a positive correlation between TMPRSS2 and the expression of HLA-I APM and IFN type I and II pathway genes (Supplementary Table 3).

### TMPRSS2-mediated upregulation of ACE2 expression

**Fig. 4:**
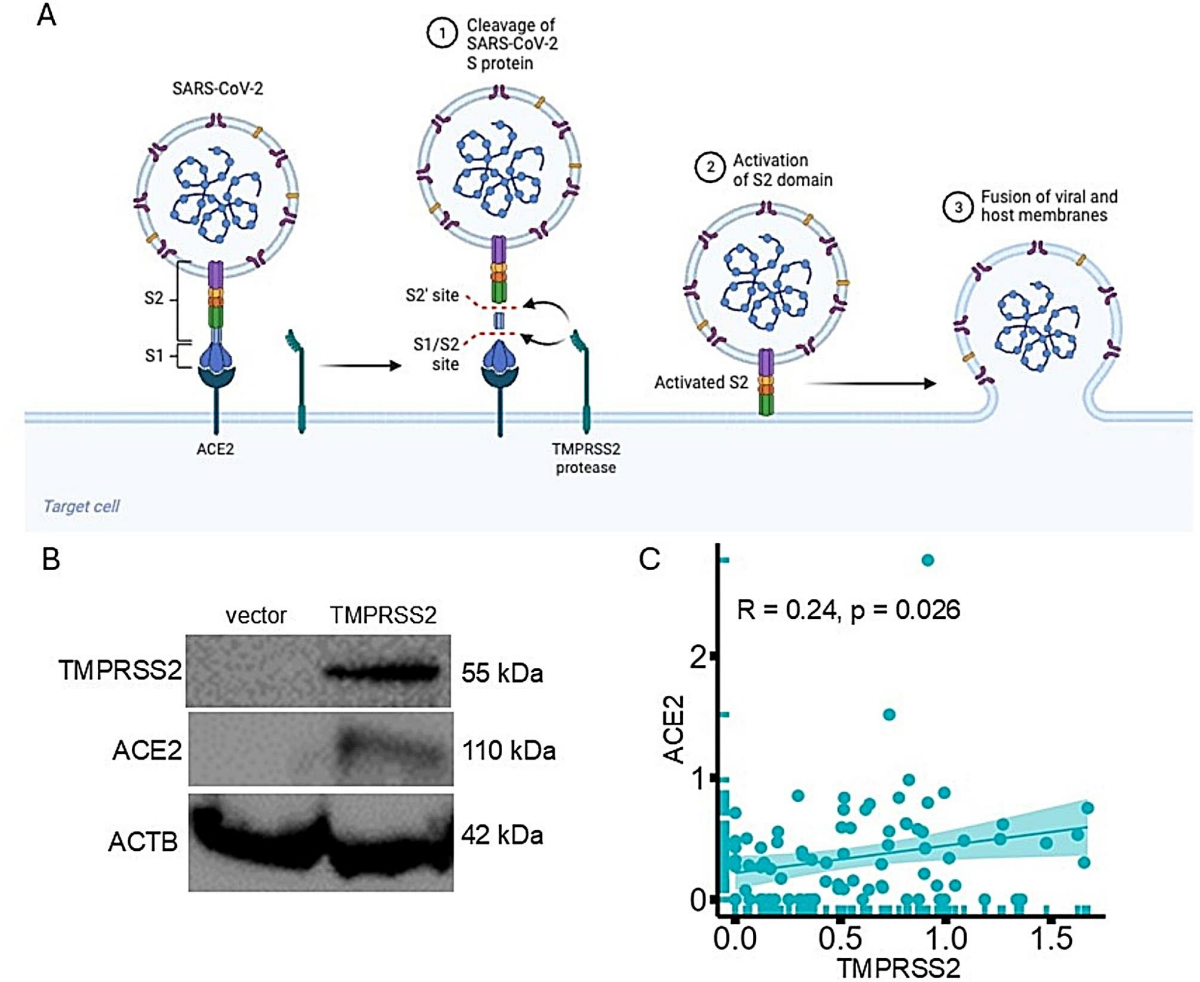
TMPRSS2-mediated upregulation of ACE2 expression. **A**: ACE2 serves as a receptor for the entry of SARS-CoV-2, while TMPRSS2 is involved in the activation of the spike (S) protein. These are crucial elements for comprehending the pathophysiology of COVID-19. **B**: ACE2 expression of TMPRSS2^high^ and mock-transfected EA.Hy926 cells was determined by Western blot using an anti-ACE2 as well as anti-TMPRSS2 mAb. **C**: A correlation plot illustrates the association of ACE2 and TMPRSS2 expression in PBMNCs of COVID-19 patients (COVID-19db)

To investigate whether there is an interplay between TMPRSS2 and ACE2, the key entry point for SARS-CoV-2 into cells (Fig. 4A), the ACE2 expression was determined in TMPRSS2^high^ vs. TMPRSS2^low^ cells. TMPRSS2^high^ EA.Hy926 cells exhibited higher protein levels of ACE2 (Fig. 4B) compared to TMPRSS2^low^ cells, suggesting a connection between TMPRSS2 and ACE2 expression in these cells. Furthermore, a correlation analysis of COVID-19 and healthy PBMNCs revealed a potential association (*r* = 0.24; *p* = 0.026) between TMPRSS2 and ACE2 (Fig. 4C). The upregulation of ACE2 by TMPRSS2 in cancer cells and PBMNCs highlights a significant overlap between cancer biology and infectious diseases, particularly in the context of the COVID-19 pandemic.

### Link of TMPRSS2 overexpression with PD-L1 surface expression

**Fig. 5:**
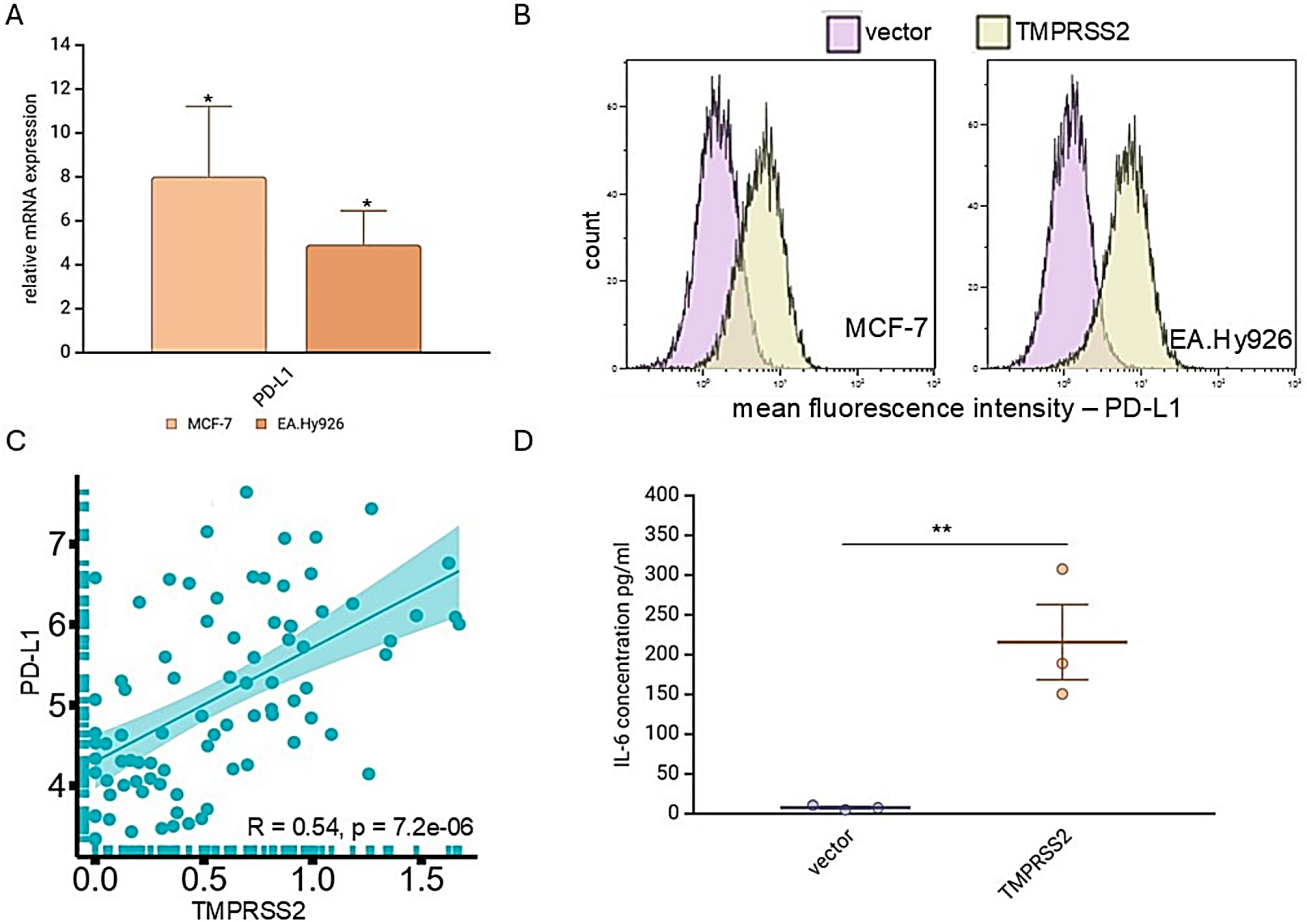
Enhanced PD-L1 and IL6 expression in TMPRSS2^high^ cells. **A**: mRNA expression by qPCR analysis was performed to assess the expression levels of PD-L1 in both TMPRSS2^high^ cells and mock-transfected control cells. **B**: Flow cytometry was used to evaluate PD-L1 surface expression in TMPRSS2 transfectants of MCF-7 and EA.Hy926 cells (*n* = 3). The results are presented as MFI. **C**: A correlation plot depicts the relationship between TMPRSS2 and PD-L1 expression in blood samples from COVID-19 patients (COVID-19db). **D**: The supernatants from TMPRSS2^high^ and TMPRSS2^low^ EA.Hy926 cells were assessed for IL-6 secretion using ELISA. The data represents the mean from three independent experiments

Since TMPRSS2 plays a significant role in immune activation and exhaustion, the expression levels of inhibitory checkpoint molecules, including PD-L1 (CD274), PD-1 (PDCD1), CTLA4 and several others (specifically ADORA2A, ADORA2B, BTLA, CYBB, HAVCR2, IDO1, LAG3, SIGLEC7, VTCN1 and CD276) was analyzed upon SARS-CoV-2 infection and TMPRSS2 overexpression (Supplementary Table 4). High levels of PD-L1 and IDO1 were found in SARS-CoV-2-infected in vitro, Calu3 cells and in vivo mouse models. These data were in line with transcriptomic analyses of TMPRSS2^high^ MCF-7 and EA.Hy926 cells demonstrated a marked increase in the expression of PD-L1 and IDO1. Furthermore, elevated PD-L1 levels in COVID-19 patients were closely linked to high mortality rates [14]. TMPRSS2^high^ MCF-7 and EA.Hy926 cells exhibited higher levels of PD-L1 mRNA and protein expression compared to TMPRSS2^low^ control cells. This was determined through qPCR (Fig. 5A), Western blot analysis (data not shown), and flow cytometry (Fig. 5B). The increased PD-L1 levels in TMPRSS2-transfected tumor cells were further supported by in silico data (COVID19db ID: COVID000010). A correlation analysis between TMPRSS2 and PD-L1 in peripheral blood mononuclear cells (PBMNCs) from COVID-19 patients and healthy controls (Fig. 5C) revealed a positive correlation (*R* = 0.54; *p* = 7.2e-08). These data suggest that the TMPRSS2^high^ cells partially resemble COVID-19 disease conditions and postulate ICPi as a treatment option.

### Increased secretion of IL-6 upon TMPRSS2 overexpression

IL-6 has been identified as the primary driver of the cytokine storm [40]. Our analysis revealed that elevated IL-6 levels were found in the supernatants of TMPRSS2^high^ cells compared to the TMPRSS2^low^ EA.Hy926 cells (Fig. 5D). These data were confirmed by in vivo mouse lung data obtained from GEO ID: GSE253635 with significantly enhanced IL-6 levels in C57BL6/J wild-type mice following SARS-CoV-2 infection [36]. Additionally, high IL-6 levels were found in PBMNC samples from patients with long COVID-19 (GEO: GSE224615; [35]) compared to uninfected PBMCs. In addition to IL-6, 15 other cytokines, chemokines, and growth factors were analyzed to provide a comprehensive overview of the inflammatory response induced by SARS-CoV-2. These include CSF2, IL-2, IL-4, IL-5, IL-1β, IL-9, IL-10, IL-12 A, IL-13, IL-17 A, IFN-γ, CCL2, CCL3, CCL4, and TNF (Supplementary Table 5). Following SARS-CoV-2 infection, C57BL6/J mice exhibited increased levels of CCL2, IFN-γ, IL-10, IL-1β and IL-6.

### Suppression of TMPRSS2 expression by a JAK2 inhibitor and small interfering RNA

**Fig. 6:**
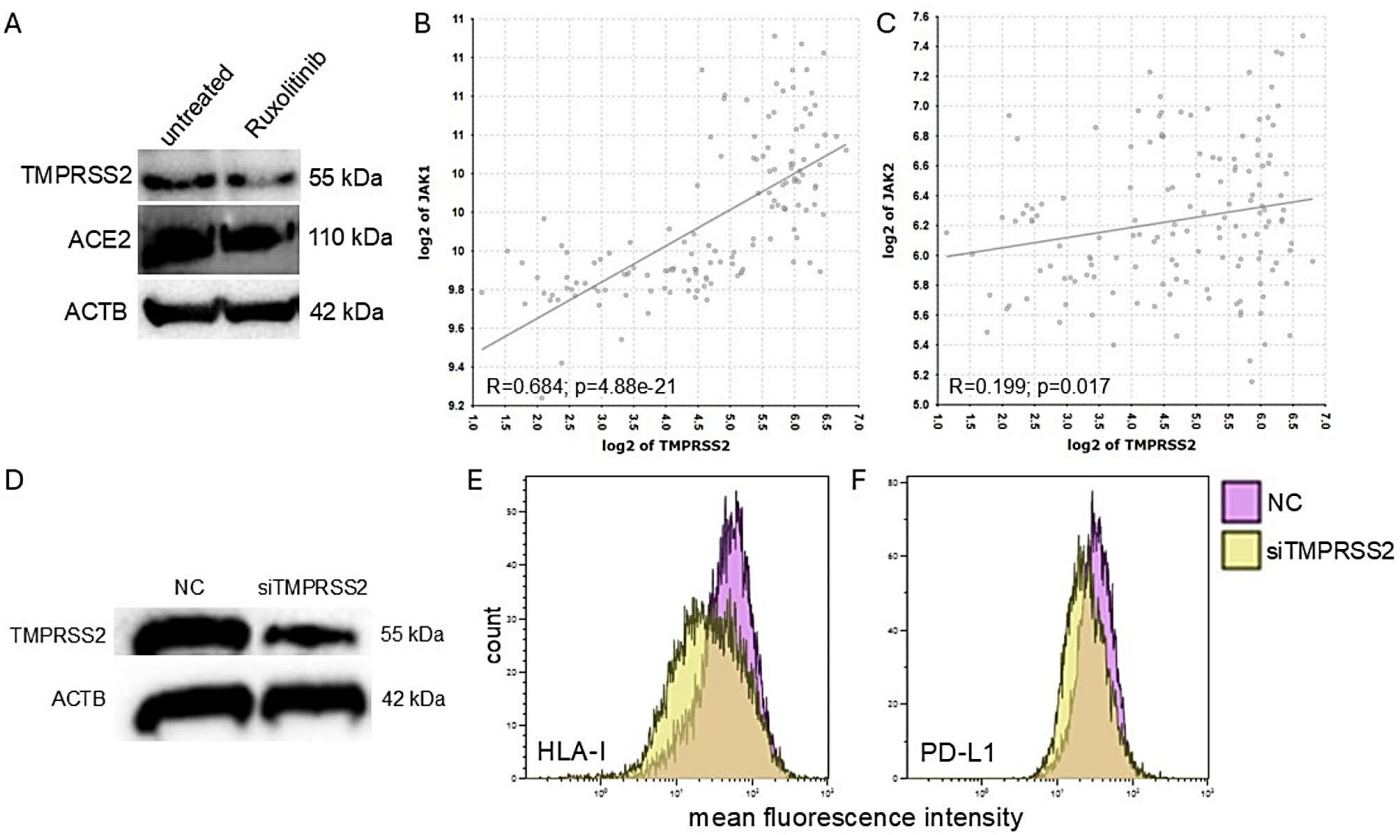
Decreased TMPRSS2 levels upon ruxolitinib treatment. **A**: Western blot analyses were performed using antibodies targeting TMPRSS2 and ACE2 in cells treated with 300 nM of ruxolitinib. **B**, **C**: Correlation plots were generated for TMPRSS2 with JAK1 (**B**) and JAK2 (**C**) using the MCF-7 dataset and R2 Genomics. **D**: Reduction of TMPRSS2 by siTMPRSS2 delivered via lipid nanoparticles was evaluated by Western blot analysis with antibodies against TMPRSS2 in MDA-MB-468 cells. **E**, **F**: Silencing of TMPRSS2 resulted in a significant reduction in HLA-I (**E**) and PD-L1 (**F**) surface expression assessed by flow cytometry

Based on the SARS-CoV-2-mediated induction of IFN pathway components, TMPRSS2^high^ MDA-MB-468 cells were treated with the JAK2 inhibitor ruxolitinib [41]. Ruxolitinib reduced JAK2 levels (data not shown) and decreased TMPRSS2 and ACE2 protein expression (Fig. 6A). Using a dataset from MCF-7 cells (GEO dataset ID: GSE21618), positive correlations of TMPRSS2 gene expression with these of JAK1 (Fig. 6B; *R* = 0.684; *p* = 4.88e-21) and JAK2 (Fig. 6C; *R* = 1.99; *p* = 0.017) were found via the R2 genomics platform.

To further explore the role of TMPRSS2, silencing experiments were conducted using siTMPRSS2. As expected, transfection with the siTMPRSS2-LNP complex decreased the TMPRSS2 protein levels in TMPRSS2^high^ MDA-MB-468 cells (Fig. 6D). This significant downregulation of TMPRSS2 correlated with a decrease of both HLA-I (Fig. 6E) and PD-L1 (Fig. 6F) surface expression, confirming a crucial role of TMPRSS2 in the modulation of both molecules (Fig. 5C).

### Increased immune cell migration of TMPRSS2high vs. TMPRSS2low MCF-7 cells after treatment with nivolumab

**Fig. 7:**
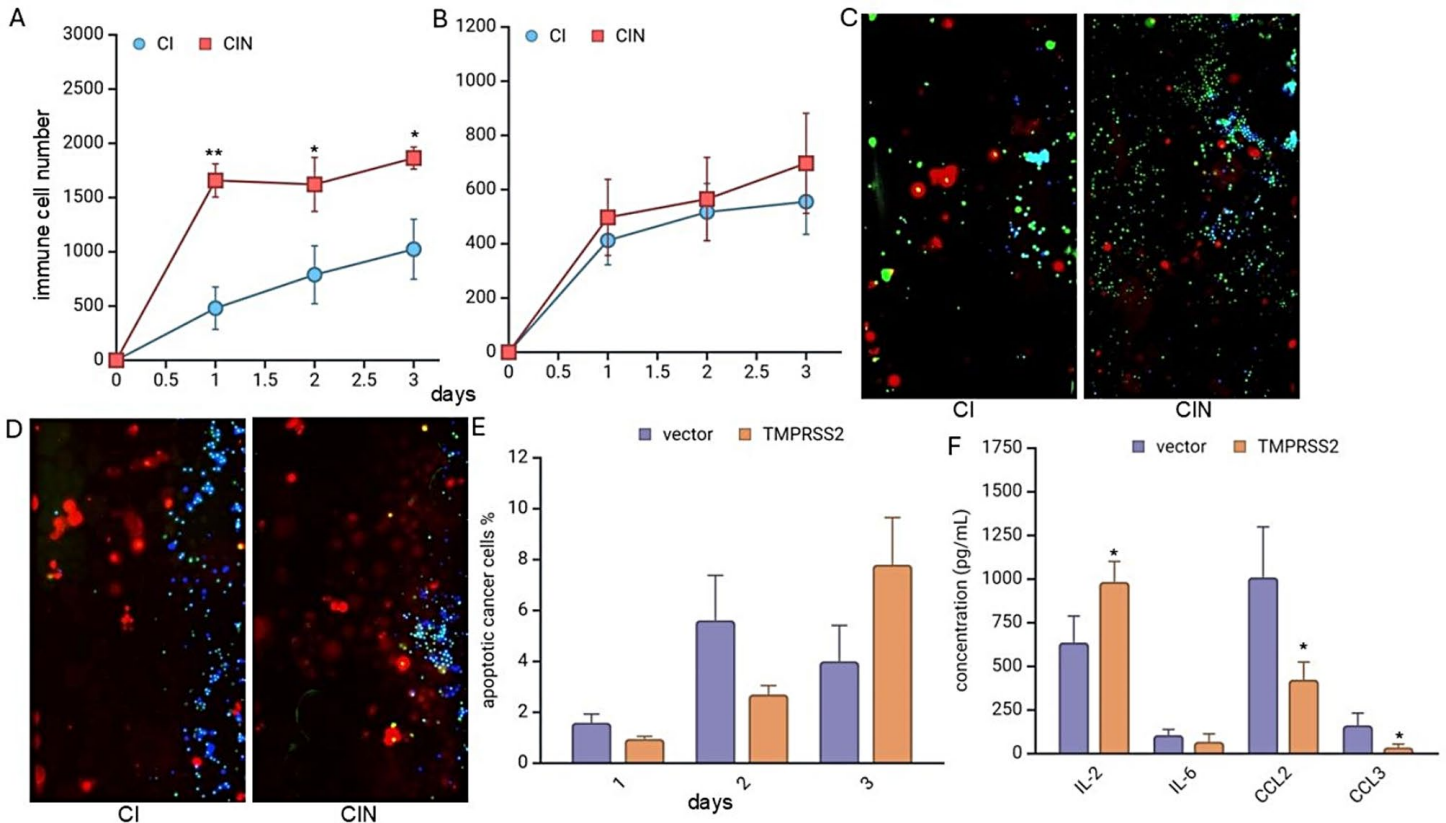
Microfluidic chip-based analyses of nivolumab-mediated effects on immune cell migration and cytokine release towards TMPRSS2^high^ cells. **A**, **B**: During a 3-day period, immune cells significantly infiltrated toward TMPRSS2^high^ MCF-7 cells (**A**), but not to TMPRSS2^low^ cells (**B**) treated with nivolumab. **C**, **D**. Representative immunofluorescent images of day 3 cancer cells, TMPRSS2^high^ (**C**) and TMPRSS2^low^ (**D**) marked in red, lymphocytes in blue and apoptotic cells in green. **E**: Percentage of apoptotic cells of TMPRSS2^high/vector^ MCF-7 cells. Nivolumab therapy on immune cells showed a marginally higher apoptosis rate in TMPRSS2^high^ compared to TMPRSS2^low^ cells. **F**: Higher release of IL-2 and reduced CCL2 and CCL3 levels in TMPRSS2^high^ MCF-7 cells when compared to mock-transfected cells. CI - Cancer cells co-cultured with Immune cells, CIN – CI treated with nivolumab

In the context of COVID-19, blocking the PD-1 pathway has been shown to counteract the immune system abnormalities induced by the virus and enhance the body’s immune response against SARS-CoV-2 [42]. To investigate whether the expression levels of TMPRSS2 may influence the efficacy of anti-PD1 mAb treatments, the immune cell migration, cancer cell proliferation and apoptosis were determined over three days of co-cultures between TMPRSS2^low^ or TMPRSS2^high^ MCF-7 and immune cells in the presence and absence of nivolumab. The immunofluorescent images captured throughout the three-day time frame post-nivolumab treatment revealed a nivolumab-mediated significant increase of the immune cell infiltration towards the TMPRSS2^high^ MCF-7 cells (Fig. 7A), but only a marginal movement of immune cells towards TMPRSS2^mock/low^ MCF-7 cells (Fig. 7B). Representative immunofluorescent images post-nivolumab treatment from immune cells targeting TMPRSS2^high^ (Fig. 7C) and TMPRSS2^low^ cells (Fig. 7D) on day 3 were shown. Additionally, the nivolumab treatment has slightly increased apoptosis rates of TMPRSS2^high^ than TMPRSS2^low^ MCF-7 cells (Fig. 7E). The nivolumab-mediated inhibition of PD-L1 on TMPRSS2^high^ MCF-7 cells might lead to increased immune cell infiltration and facilitate T cell activation. These data suggest the therapeutic potential of this treatment in redirecting the immune response against these cancer cells.

### Link of altered cytokine release and TMPRSS2 expression upon nivolumab treatment

The impact of nivolumab on the cytokine release in the cell supernatants during the co-culture of PBMNCs with TMPRSS2^high^ and TMPRSS2^low^ MCF-7 cells was investigated using a human FirePlex^®^-96 key cytokine immunoassay panel. As shown in Fig. 7F, treatment of TMPRSS2^high^ and TMPRSS2^low^ MCF-7 cells with nivolumab decreased the release of CCL2 and CCL3. However, there was an increase in IL-2 secretion in the TMPRSS2^high^ cells compared to the TMPRSS2^low^ cells. It’s important to note that IL-2 was added during the experiment, which may have influenced its secretion. The elevated IL-6 secretion (Fig. 5E) was slightly but not significantly reduced in the presence of TMPRSS2^high^ cells upon nivolumab (Fig. 7F). No statistically significant changes of other cytokines were detected in the supernatants of untreated and nivolumab-treated TMPRSS2^high^ cells suggesting no interference of ICPi with the cytokine storm (Supplementary Table 6).

## Discussion

**Fig. 8:**
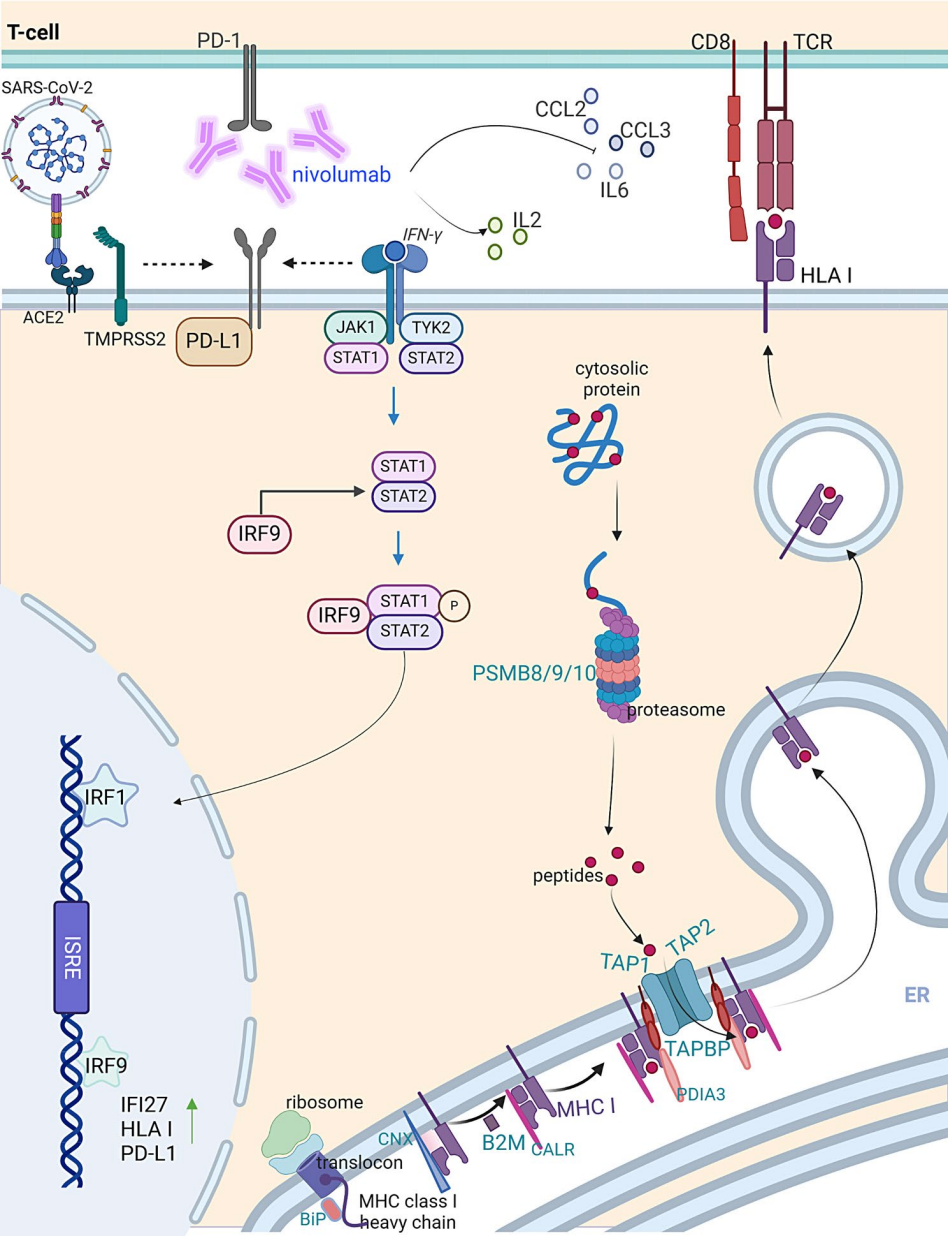
Cells exhibiting elevated levels of TMPRSS2 demonstrated increased levels of PD-L1, the IFN pathway, HLA class I antigen presentation machinery (APM) and immune responses in comparison to those with lower TMPRSS2 levels. This indicates that the expression of TMPRSS2 across various model systems and following SARS-CoV-2 infection correlates with alterations in host immunogenicity. These alterations may influence the efficacy of the PD-1 inhibitor nivolumab, which enhances the migration of immune cells and decreases cytokines associated with innate immunity (created with BioRender.com)

Patients with cancer are more affected by COVID-19 with longer hospitalization time and increased mortality [8, 43]. So far, the impact of additional treatments affecting the immune system, such as immunotherapy or immunosuppressive agents, on the severity of COVID-19 in tumor patients has not yet been determined [8]. Our study showed that TMPRSS2 affected the expression of the IFN signaling components including JAK-STAT genes in both tumor cell lines and patients’ samples, which is consistent with the COVID-19 genome databases (COVID19db; GEO: GSE147507). This is linked to an increased HLA-I and PD-L1 expression suggesting that cancer patients with COVID-19 might respond more to ICPi treatments directed against the PD1/PD-L1 axis (Fig. 8).

Since coronaviruses are considered emerging and re-emerging pathogens [44], anti-viral strategies targeting multiple coronaviruses are urgently needed. High TMPRSS2 expression levels associated with SARS-CoV-2 infection were also frequently found in various cancers. Despite TMPRSS2 as well as ACE2 has higher expression levels after the onset of COVID-19 and cancers [45], no detailed study is available on their expression and interaction in cancer patients infected with COVID-19 or any other coronaviruses. Recently, a correlation between ACE2 expression and immune response has been reported [46], which highlights the potential role of coronavirus target genes in shaping the tumor microenvironment (TME) and influencing tumor behavior during viral infections. The in vitro tumor models investigated in this study showed that TMPRSS2 could enhance ACE2 expression in cancer cells, but an in depth understanding of the precise molecular pathways, through which TMPRSS2 regulates ACE2, requires further investigations.

There exists evidence of an IFN-γ component-mediated control of APM molecules in tumors [47], but APM components have not been extensively studied in the context of COVID-19. Interestingly, an upregulation of many APM components, particularly TAP2, was found in TMPRSS2^high^ cells and COVID-19 PBMNCs. Furthermore, KEGG pathway analyses indicated an enrichment of several signaling pathways associated with virus infection and the NOD-like receptor signaling in PBMNCs of patients with COVID-19 disease [48, 49]. In a multi-cohort study, IFI27 transcription was identified as an early predictor for the outcome of COVID-19 patients [50]. Consistent with these data, our TMPRSS2^high^ EA.Hy926 transfectants showed increased IFI27 mRNA (Log2FC 3.57; p 3.68E-15) and protein levels. IFNs play a dual role in immune responses. They enhance the immune system’s ability to eliminate tumor cells, while promoting mechanisms leading to inflammation and allowing immune evasion by e.g. upregulation of PD-L1. The IFN-mediated increase in PD-L1 expression represents a form of “adaptive resistance” [51], which we demonstrated in our results with an upregulation of IFN and PD-L1 in cells with high TMPRSS2 expression and in COVID-19 patients. Furthermore, increased expression of IFN signaling pathway molecules along with IL-6 in long COVID-19 patients indicated a potential role of IFN signaling in the prolonged effects in these patients [35]. Upon binding of IFN to its receptor, JAK kinases are activated leading to STAT1 phosphorylation [52] and consequently to induction of PD-L1 expression [53]. Interestingly, TMPRSS2^high^ cells expressed high levels of phosphorylated STAT1 thereby triggering PD-L1 surface expression. An improved understanding of the connection between PD-L1 expression and JAK-STAT signaling might provide novel options for combination therapies. The efficacy of PD-1/PD-L1 blockade could be enhanced by the use of JAK inhibitors [51]. Indeed, in patients with Hodgkin lymphoma [54] and in those with non-small cell lung cancer [55], JAK inhibition improved checkpoint blockade immunotherapy. Additionally, JAK inhibitors, such as ruxolitinib, have been discussed for their potential in treating COVID-19 [24, 56] with focus on ACE2. This was extended by our study to TMPRSS2, which is a direct target of ruxolitinib. Elevated levels of the ICP molecule IDO1, alongside PD-L1, in the transcriptome profile of TMPRSS2^high^ and SARS-CoV-2-infected cells were found suggesting exploring the potential application of IDO1 inhibitors.

Growth factors and inflammatory cytokines, such as EGF, IL-6, IFN-γ, TNF-α and TGF-β, can induce PD-L1 expression [57, 58]. However, whether anti-PD-1/PD-L1 treatment would be beneficial for COVID-19 patients has to be investigated, since this treatment might enhance the cytokine storm associated with higher COVID-19 morbidity and mortality [59, 60]. It is noteworthy that melanoma patients with COVID-19 displayed better outcomes when treated with ICPi [61, 62]. Therefore, anti-PD-1 therapy may also benefit SARS-CoV-2-infected patients. Our data indicates that TMPRSS2^high^ cells, which have increased PD-L1 expression, responded to nivolumab, demonstrating a TMPRSS2-dependent response along with increased immune cell infiltration into cancer cells.

Cytokines mediate the expansion, activation and trafficking of effector lymphocytes, but can also recruit regulatory T cells [63]. Additionally, TMPRSS2-mediated higher STAT1 expression could regulate IFN-γ-induced CCL2 expression [64]. This decline in CCL2 and CCL3 might revert T cell exclusion and the cytokine storm thereby affecting COVID-19 severity. A decreased innate immunity and the release of anti-inflammatory cytokines CCL2 and CCL3, respectively, were detected in TMPRSS2 transfectants co-cultured with PBMNCs upon nivolumab treatment. Targeting TMPRSS2 with ruxolitinib may reduce its expression and revert the associated immune suppression, while nivolumab could enhance immune cell activity against tumors expressing TMPRSS2. A recent clinical trial demonstrated that ruxolitinib in combination with nivolumab improved the clinical outcome of patients primarily not responding to checkpoint blockade. This result paves the way for novel therapeutic strategies for cancer and COVID-19 patients [54]. On the other hand, further studies are needed to understand how anti-PD-1 therapy affects TMPRSS2, cytokine storm, IFN signaling and lymphocyte composition and function following SARS-CoV-2 infection.

## Conclusions

Our research indicates a link between TMPRSS2 and different types of tumors as well as an effect of SARS-CoV-2 infection on the immune response by activating immune-related pathways. Targeting TMPRSS2 as a universal coronavirus strategy shows potential for the design of novel treatments and vaccines that can offer broad protection against current and future coronavirus threats. Our study emphasizes the importance of using in vitro disease models and RNA-seq datasets to form hypotheses relevant to human disease. Our findings show that treatment with an anti-PD-1 antibody improved the infiltration of immune cells and inhibited the production of inflammatory cytokines in an in vitro TMPRSS2^high^ model. These results suggest that anti-PD-L1 treatment could restrict T cell exhaustion and hinder virus infectivity at an early stage of virus entry. Combining TMPRSS2 inhibitors with immune modulators, such as PD-1 inhibitors and/or JAK inhibitors, may offer a comprehensive approach for the treatment of COVID-19, but the therapeutic strategies need to be balanced between enhancing anti-viral immunity and preventing excessive inflammation and activation of the immune system, which can cause tissue damage and worsen outcomes as observed in severe COVID-19 cases.

## Electronic supplementary material

Below is the link to the electronic supplementary material.

Supplementary Material 1

## Data Availability

All data generated or analyzed during this study are included either in this article or in the supplementary information files. The datasets generated during and/or analyzed during the current study are available from the corresponding author upon reasonable request.

## Abbreviations

2D: Two dimensional
3D: Three dimensional
Ab: Antibody
ACE2: Angiotensin-converting enzyme-2
APM: Antigen processing machinery
ATCC: American Type Culture Collection
BC: Breast cancer
β2-m: β2-microglobulin
BP: Biological process
BC: Breast cancer
CC: Cellular component
CoV: Coronavirus
CRC: Colorectal carcinoma
CSF: Colony stimulating factor
DEG: Differentially expressed gene
DGE: Differential gene expression
DSS: Disease-specific survival
FCS: Fetal caf serum
FDR: False discovery rate
GADPH: Glyceride aldehyd-3-phosphate dehydrogenase
GO: Gene ontology
HC: Heavy chain
HLA: Human leukocyte antigen
HRP: Horse reddish peroxidase
ICP: Immune checkpoint
ICPi: Immune checkpoint inhibitor
IDO: Indoleamine 2,3-dioxygenase
IFN: Interferon
IL: Interleukin
KEGG: Kyoto Encyclopedia of Genes and Genomes
LMP: Low molecular weight protein
LNP: Lipid nanoparticles
mAb: monoclonal antibody
MAS: Macrophage activation syndrome
MCP-1: Monocyte chemoattractant protein-1
MF: Molecular function
MFI: Mean fluorescence intensity
MHC: Major histocompatibility complex
MIP-1α: Macrophage inflammatory protein 1 alpha
NK: Natural killer
OS: Overall survival
PBMC: Peripheral blood mononuclear cells
PD1: Programed death receptor 1
PD-L1: Programed death ligand 1
S: Spike
SARS: Severe acute respiratory syndrome
TAP: Transporter associated with antigen processing
TCGA: The Cancer Genome Atlas
TGF-β: transforming growth factorβ
TME: Tumor microenvironment
TMPRSS2: Transmembrane protease, serine 2TNF, tumor necrosis factor
TNF: Tumor necrosis factor
TAPBP: Tapasin
