# Supplementary material for: Link of TMPRSS2 expression with tumor immunogenicity and response to immune checkpoint inhibitors in cancers
Source: J Transl Med. 2025 Mar 7;23:294. doi: 10.1186/s12967-025-06177-z (PMC11887338; doi:10.1186/s12967-025-06177-z)
Supplement: Supplementary file 1 — Supplementary Material 1 [file 12967_2025_6177_MOESM1_ESM.pdf]

Supplementary Figure 1: Comparison of transcriptional profiles in MCF-7 and EA.Hy926 cells with TMPRSS2^{high} and TMPRSS2^{low} groups using RNA sequencing.

A: Volcano plot illustrating the differentially expressed genes (DEGs) between the TMPRSS2^{high} and TMPRSS2^{low} MCF-7 groups.

B: Volcano plot illustrating the DEGs between the TMPRSS2^{high} and TMPRSS2^{low} EA.Hy926 groups.

Genes that are significantly downregulated are indicated in red, significantly upregulated genes in green and genes with non-significant regulation in blue.

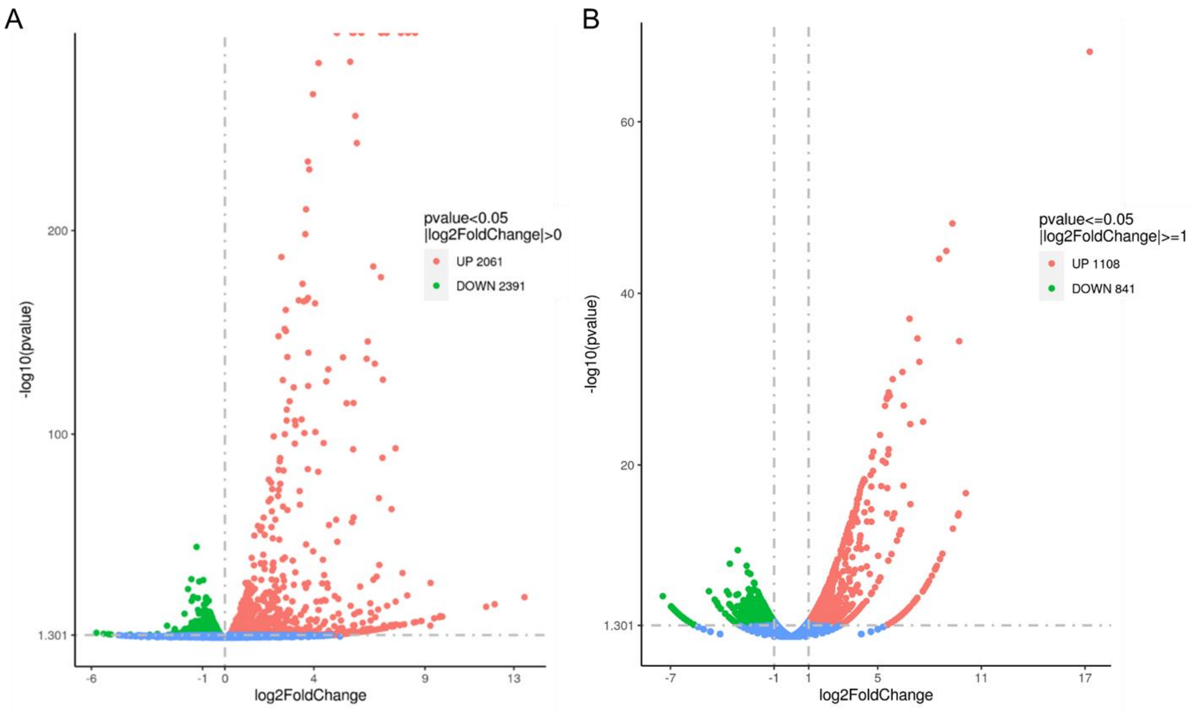

Supplementary Figure 2: The top 20 enriched KEGG terms from downregulated genes of TMPRSS2^{high} and TMPRSS2^{low} MCF-7 and EA.Hy926 cells and COVID-19 PBMNC.

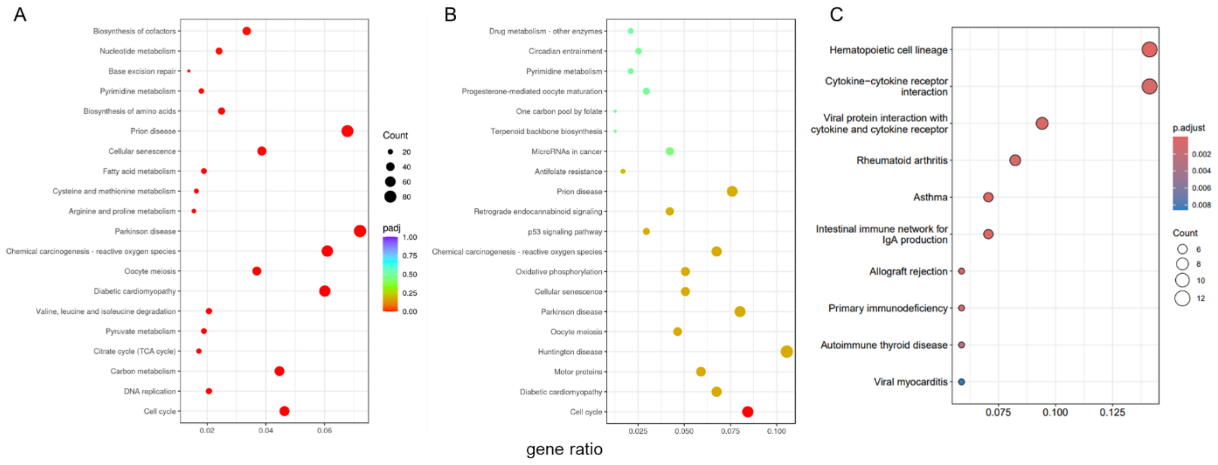

Supplementary Table 1: Primers employed for qPCR evaluations.

gene	Forward	reverse
TMPRSS2	ACTCTGGAAGTTCATGGGCAG	TGAAGTTTGGTCCGTAGAGGC
HLA-ABC	GCCTACCACGGCAAGGATTAC	GGTGGCCTCATGGTCAGAGA
PD-L1	GAAGTCATCTGGACAAGCAGTG	CAGAGGTAGTTCTGGGATGACC
TAP1	GGAATCTCTGGCAAAGTCCA	TGGGTGAACTGCATCTGGTA
TAP2	CCAAGACGTCTCCTTTGCAT	TTCATCCAGCAGCACCTGTC
IRF1	GCAGCTACACAGTTCCAGG	GTCCTCAGGTAATTTCCCTTCCT
IRF2	TGCGGTCCTTGACTTCAACTA	ATATTCCTCTTCCGCCAGTG
IRF9	GTCCAGCTGTCTGGAAGACT	TCCTCTTCCTCCTTCCTCTC
JAK1	CCACTACCGGATGAGGTTCTA	GGGTCTCGAATAGGAGCCAG
TYK2	GAGATGCAAGCCTGATGCTAT	GGTTCCCGAGGATTCATGCC
STAT1	CAGCTTGACTCAAAATTCCTGGA	TGAAGATTACGCTTGCTTTTCCT
STAT2	GAGCCAGCAACATGAGATTGA	GCCTGGATCTTATATCGGAAGCA
STAT3	ATCACGCCTTCTACAGACTGC	CATCCTGGAGATTCTCTACCACT
STAT6	CGAGTAGGGGAGATCCACCTT	GCAGGAGTTTCTATCAAGCTGTG
STING	GTCTCCAAGCCTCTGGACTG	TGTCAAGTCCTGGACCCTTC
CCSER2	GACAGGAGCATTACCACCTCAG	CTTCTGAGCCTGGAAAAAGGGC
ACTB	ACTCTTCCAGCCTTCCTTCC	AGCACTGTGTTGGCGTACAG
GAPDH	CTGGTAAAGTGGATATTGTTGCCAT	TGGAATCATATTGGAACATGTAAACC

Supplementary Table 2: Analysis of up-regulated genes in TMPRSS2^{high} MCF-7 and EA.Hy926 cells under SARS-CoV-2 infection in an organoid model.

Datasets were obtained from GEO, including an organoid model infected with the SARS-CoV-2 Alpha variant (B.1.1.7) (GEO: GSE178333).

gene	log2FC	p-value
HLA-B	0.36	0.08
IFI27	4.54	0.00
IFI44L	4.00	0.01
IFI6	3.21	0.00
IFIT1	4.01	0.01
IFIT2	2.20	0.02
IFIT3	1.99	0.01
IFNB1	NA	0.08
IL6	NA	NA
IRF1	0.16	0.35
IRF9	0.90	0.02
ISG15	3.77	0.01
LGALS3BP	0.18	0.29
MYD88	-0.18	0.28
NFKBIA	-0.34	0.20
OAS1	1.55	0.03
OAS2	4.56	0.02
OAS3	1.38	0.00
OASL	3.75	0.00
STAT1	0.57	0.06
STAT2	0.12	0.36
TNFAIP3	0.15	0.38

Supplementary Table 3: Correlation of TMPRSS2 expression with HLA class I and APM components and IFN pathway components were performed using R2 Genomics (http://r2.amc.nl) and two different cancer datasets, Tumor Breast Invasive Carcinoma - TCGA – 782 and Mixed Tumor Lung - GSE18842 – 91.

gene	Tumor Breast Invasive Carcinoma		Mixed Tumor Lung	
	r-value	p-value	r-value	p-value
HLA-A	0.117	1.10e-03	0.281	6.98E-03
HLA-B	0.169	1.98e-06	0.374	2.61E-04
HLA-C	0.107	2.73e-03	0.414	4.50E-05
HLA-E	0.231	5.97e-11	0.53	6.58E-08
HLA-G	0.262	1.07e-13	0.26	0.013
TAP1	-0.002	0.949	0.15	0.156
TAP2	0.198	2.17e-08	0.219	0.037
TAPBP	0.151	2.30e-05	0.386	1.59E-04
CALR	0.019	0.587	-0.145	0.171
CANX	0.032	0.365	-0.238	0.023
ERAP1	0.159	7.78e-06	0.253	0.016
ERAP2	0.353	2.24e-24	0.165	0.119
PSMB9	0.128	3.24e-04	0.331	1.34E-03
PSMB8	0.065	0.068	0.308	2.99E-03
PSMB10	0.017	0.639	0.469	2.79E-06
CD274	0.123	5.72e-04	0.329	1.45E-03
B2M	0.119	8.52e-04	0.304	3.41E-03
IFNG	0.183	2.54e-07	0.271	9.42E-03
IFNGR1	-0.007	0.835	0.546	2.18E-08
IFNGR2	-0.022	0.539	0.291	5.18E-03
JAK1	-0.023	0.521	0.41	5.48E-05
JAK2	0.012	0.738	0.466	3.30E-06
STAT1	0.062	0.083	-0.062	0.558
IRF1	0.166	3.15e-06	0.579	1.88E-09

Supplementary Table 4: A transcriptomic analysis of inhibitory checkpoint molecules following SARS-CoV-2 infection and the overexpression of TMPRSS2.

gene	after SARS-CoV-2 infection					
	Calu3		blood samples		mouse model	
	Log2FC	p-value	Log2FC	p-value	Log2FC	p-value
ADORA2A	1.932	0.001	-0.147	0.110	1.17761	0.00526
ADORA2B	0.214	0.103	0.022	0.887	0.91125	0.00362
BTLA	0.395	0.187	-0.674	0.000	-0.0803	0.3451
CD274	4.963	0.000	1.190	0.000	3.21695	0.0013
CTLA4	0.120	0.398	-0.300	0.095	1.46657	0.00096
CYBB	-1.114	0.021	0.354	0.003	1.64942	3.8E-05
HAVCR2	-0.148	0.380	0.415	0.001	1.12298	0.03453
IDO1	4.046	0.000	-0.696	0.000	2.62049	0.0128
LAG3	0.000	NA	0.440	0.056	1.62118	0.01464
PDCD1	0.545	0.183	-0.252	0.142	3.55459	0.04154
SIGLEC7	0.000	NA	-0.112	0.407	NA	NA
VTCN1	-0.016	0.431	0.300	0.032	-0.197	0.24456
CD276	-0.226	0.049	0.210	0.039	0.96263	0.02653

gene	after TMPRSS2 overexpression			
	MCF-7		EA.Hy926	
	Log2FC	p-value	Log2FC	p-value
ADORA2A	0.874	0.309	-0.651	0.776
ADORA2B	-0.580	0.069	0.399	0.362
BTLA	NA	NA	NA	NA
CD274	4.213	0.000	1.381	0.001
CTLA4	0.005	0.996	NA	NA
CYBB	0.635	0.570	NA	NA
HAVCR2	2.983	0.220	1.161	0.206
IDO1	8.066	0.000	5.793	0.023
LAG3	0.923	0.193	-1.324	0.026
PDCD1	-0.981	0.785	NA	NA
SIGLEC7	NA	NA	4.049	0.510
VTCN1	1.203	0.000	NA	NA
CD276	-0.080	0.416	0.791	0.057

Supplementary Table 5: Gene expression analysis in C57BL6/J wild-type mice following day 2 of SARS-CoV-2 MA10 infection (GEO ID: GSE253635)

gene	log2FC	p-value
Ccl2	5.38	0.03
Ccl3	4.05	0.12
Ccl4	4.29	0.08
Csf2	1.61	0.01
Ifng	2.99	0.05
Il10	4.95	0.02
Il12a	-0.15	0.42
Il13	-0.14	0.44
Il17a	#DIV/0!	#DIV/0!
Il1b	3.14	0.02
Il2	-1.66	0.13
Il4	-1.53	0.07
Il5	0.40	0.19
Il6	6.43	0.01
Il9	#DIV/0!	#DIV/0!
Tnf	2.33	0.07

Supplementary Table 6: Microfluidic chip-based analysis of cytokine release with TMPRSS2$^{high/low}$ cells upon nivolumab treatment.

cytokines	TMPRSS2 low	TMPRSS2 high	TMPRSS2 high vs. low
	mean		p value
CSF2	7.97	8.37	0.45
IFNG	20.05	39.09	0.12
IL1B	8.38	7.80	0.45
IL2	634.32	981.15	0.05
IL4	0.76	1.29	0.09
IL5	0.39	0.49	0.22
IL6	104.06	65.57	0.25
CXCL8	3034.83	2673.32	0.19
IL9	1.81	2.99	0.15
IL10	5.08	4.04	0.11
IL12A	1.51	3.25	0.01
IL13	6.53	15.49	0.02
IL17A	2.38	0.77	0.22
CCL2	1007.35	421.03	0.04
CCL3	159.59	33.37	0.05
CCL4	350.92	116.63	0.08
TNF	30.79	30.66	0.50
